# Stage-Related Alterations in Renal Cell Carcinoma – Comprehensive Quantitative Analysis by 2D-DIGE and Protein Network Analysis

**DOI:** 10.1371/journal.pone.0021867

**Published:** 2011-07-07

**Authors:** Heike Junker, Simone Venz, Uwe Zimmermann, Andrea Thiele, Christian Scharf, Reinhard Walther

**Affiliations:** 1 Department of Medical Biochemistry and Molecular Biology, University of Greifswald, Greifswald, Germany; 2 Interfacultary Institute of Genetics and Functional Genomics, University of Greifswald, Greifswald, Germany; 3 Department of Urology, University of Greifswald, Greifswald, Germany; 4 Department of Pathology, University of Greifswald, Greifswald, Germany; 5 Department of Otorhinolaryngology, Head and Neck Surgery, University of Greifswald, Greifswald, Germany; Faculdade de Medicina, Universidade de São Paulo, Brazil

## Abstract

Renal cell carcinoma accounts for about 3% of adult malignancies and 85% of neoplasms arising from the kidney. To identify potential progression markers for kidney cancer we examined non-neoplastic and neoplastic kidney tissue from three groups of patients, which represent different tumor stages (pT1, pT2, pT3) by a fluorescence two-dimensional difference gel electrophoresis (2D-DIGE) approach combined with MALDI-ToF-MS/MS. Delta2D software package was used for gel image based quantification and statistical analysis. Thereby, a comprehensive Principal Component Analysis (PCA) could be performed and allowed a robust quality control of the experiment as well as a classification of the analyzed samples, which correlated with the predicted stages from the pathological examination. Additionally for selected candidate proteins we detected a correlation to the tumor grading as revealed by immunohistochemistry. On the 2D protein map 176 spots out of 989 were detected as at least 2-fold differentially expressed. These spots were analyzed by MALDI-ToF-MS/MS and 187 different proteins were identified. The functional clustering of the identified proteins revealed ten groups. Within these groups we found 86 enzymes, 63 proteins of unknown function, 14 transporter, 8 peptidases and 7 kinases. From the systems biology approach we could map many of these proteins in major pathways involved in remodelling of cytoskeleton, mitochondrial dysfunctions and changes in lipid metabolism. Due to complexity of the highly interconnected pathway network, further expression and functional validation of these proteins might provide new insights in kidney cancer progression to design novel diagnostic and therapeutic strategies.

## Introduction

Renal cell carcinoma is the most common cancer of the kidney and accounts for about 209 000 new cases per year worldwide and 102 000 deaths [1]. The curable therapy is a partial or total nephrectomy and the clinical outcome depends on tumor stage and grading. Unfortunately, about 20% of patients already show metastases at diagnosis and additionally about 30% develop metastases following surgery. In consequence of resistance against chemotherapeutic agents and radiation therapy the therapeutic options for these patients are very limited. Although rapid progress has been made to understand tumorigenesis in renal cell carcinomas and other tumor entities, the primary events leading to dysfunction of the control of proliferation remain unclear. Studies of genes involved in cell cycle control and even array analysis studying the global gene expression pattern of neoplastic cells in comparison with non-neoplastic cells led to the identification of differentially expressed genes [2]. Using the rendered gene expression pattern several research groups identified differentially expressed genes, which could be used to discriminate between non-neoplastic and neoplastic tissue and which also correlated to the histological classification of the tumor or even with the clinical outcome [3]–[5]. Considering that regulatory dysfunctions can be caused not only by altered gene expression but also by posttranslational modifications of proteins, analyses of the proteome are of special importance to detect disease-associated protein alterations [6]. Recently we and others used these methods to identify differentially expressed proteins comparing non-neoplastic and neoplastic kidney tissues [7]–[13].

Taking into account that tumor progression is reflected by characteristic consecutive tumor stages we now studied neoplastic tissues from kidneys belonging to different tumor stages and gradings as well as non-neoplastic specimens, which may allow the identification of progress markers or such of prognostic value.

Two-dimensional polyacrylamide gel electrophoresis (2D-PAGE), a powerful tool in proteomics used for protein separation and expression profiling is one of the core technologies used in proteomics [14], [15]. Protein expression analysis could lead to the characterization of molecular events associated with cancer. The successful management of human malignancies requires diagnostic and prognostic methods as well as identification of therapeutical targets. Proteome analysis may be able to serve as a tool that provides useful information for all [16].

In the present study, we investigated the stage-related protein alterations in kidney cancer from defined pathological characterized samples using 2D-DIGE [17] and MALDI-ToF-MS/MS for protein identification. The differentially expressed proteins were analyzed by Ingenuity Systems. Systems biology network analysis might provide information about the potential role of proteins in different regulatory pathways controlling kidney cancer progression and might predict new biomarkers for further validation using Western blotting and immunohistochemical (IHC) approaches.

## Materials and Methods

### Ethics statement

The study was based on renal carcinoma patients diagnosed and treated at the Department of Urology, University of Greifswald. Tissue samples and patients data for this study were obtained and used after ethics approval from the Ethics Committee at the Medical Faculty of the University of Greifswald (III UV 12/03) and in accordance with the declaration of Helsinki. We obtained written informed consent of all participants.

### Patient material

Representative samples from non-neoplastic and neoplastic tissues were obtained immediately after nephrectomy. Kidneys were sliced longitudinally along the largest diameter of the tumor. From one half tissue samples were taken from the tumor and from macroscopically non-neoplastic surrounding renal cortex tissue, frozen in liquid nitrogen and stored at −80°C until use. From the second half corresponding samples were fixed in formalin and embedded in paraffin for subsequent immunohistochemistry and examination by a pathologist. Furthermore, the pathological staging was determined according to the latest TNM-classification [18] and grading according to Fuhrman [19]. We analyzed in the 2D-DIGE experiment one control and three tumor groups with different stagings: pT1, pT2, pT3. Each group consisted of 9 samples. A series of paraffin sections from 22 different samples containing tumor sample with different gradings (7×G1, 7×G2 and 8×G3) were subjected to immunohistochemistry.

### Protein isolation and labeling with CyDyes

The preparation of protein samples was performed with the TRIzol^®^Reagent (Invitrogen, Karlsruhe, Germany) according to the manufacturer's protocol. Finally, the vacuum-dried protein pellet was dissolved in sample buffer (8 M urea, 2 M thiourea, 4% CHAPS, 65 mM dithiothreitol, 40 mM Tris). Protein amounts were quantified by the Bradford method [20]. The quality of the samples for 2D-DIGE was evaluated by mini 2D-PAGE (7 cm IPG strips). The protein lysates were labeled with CyDyes according to the manufacturer's protocol for minimal labeling (CyDye DIGE Fluor minimal dyes, GE Healthcare, Munich, Germany). In order to minimize dye-specific labeling artefacts, Cy3 and Cy5-labeling patterns were swapped among the same group of samples. An internal standard pool with equal amounts of each protein sample (25 µg) was used to reduce inter-gel variation. The pooled internal standards were labeled with Cy2. 50 µg protein of each sample was labeled with 400 pmol of corresponding dye on ice in the dark for 30 min. Reaction was quenched with 10 mM L-lysine for 10 min under the same conditions.

### Two-dimensional polyacrylamide gel electrophoresis (2D-PAGE)

Samples were applied to IPG strips (pH 3-10 NL, 24 cm, GE Healthcare, Munich, Germany) by in-gel rehydration. For DIGE-gels a total amount of 150 µg protein and for preparative gels a total of 700 µg protein were filled to a volume of 450 µl with rehydration buffer (8 M urea, 2 M thiourea, 2% CHAPS, 15 mM dithiothreitol and 0.5% IPG-buffer pH 3-10 NL [GE Healthcare, Munich, Germany]). After rehydration overnight, isoelectric focusing (IEF) was carried out at 20°C on a PROTEAN IEF Cell (Bio-Rad, Munich, Germany) for a total of 55 000 Vhr. Subsequently, the IPG strips were stored at -20°C or immediately soaked twice in a solution containing 6 M urea, 2% SDS, 375 mM Tris-HCl (pH 8.8), 20% glycerol and 1% dithiothreitol for the first, or 2.5% iodoacetamide for the second step of equilibration. Strips were placed on vertical SDS-PAGE gels and fixed with 1% agarose. As tracking dye a few grains of bromophenol blue were added. SDS-PAGE was carried out using the PROTEAN plus Dodeca Cell (Bio-Rad, Munich, Germany), with gels of 1.5 mm thickness and an acrylamide concentration of 12.5%. Polyacrylamide gels for DIGE were cast in low fluorescence glass plates. Electrophoresis was performed with constant voltage (80 V) at 20°C until the dye front reached the bottom of the gel. The complete device is protected from light. After electrophoresis DIGE gel cassettes were washed with ddH_2_O and wiped with dust free tissue paper. The Cy2 (Internal standard), Cy3 and Cy5 labeled proteins in each gel were visualized by using a Typhoon 9400TM laser scanner (GE Healthcare) at 100 microns by using different excitation and emission wavelengths directly from gels between glass plates. Optimal excitation/emission wavelengths for fluorescence detection are 488/520 nm for Cy2, 532/580 nm for Cy3, and 633/680 nm for Cy5. For preparative gels: 50 µg protein was labeled by Cy2 (as internal control for gel matching) and mixed with 650 µg unlabeled protein. After 2D separation the gel was scanned using a Typhoon 9400TM laser scanner and subsequently stained with Roti^®^-Blue (Carl Roth GmbH, Karlsruhe, Germany), a colloidal coomassie brilliant blue G250 stain. Briefly, gels were fixed in 40% methanol, 15% acetic acid for at least 4 hrs and then immersed in colloidal staining solution overnight. To remove background staining gels were washed in 20% methanol.

Each sample was run in duplicate implementing a Cy3/Cy5 dye swap thus enabling correction of dye-specific effects in the subsequent statistical analysis.

### Software and bioinformatic approaches

2D-gel image analysis was performed using Delta2D Software version 4.0.8 (DECODON GmbH, Greifswald, Germany).

All gel images were matched with the Delta2D software and a synthetic fusion gel was prepared using a union fusing option. Final spot detection was performed on the fused gel.

The resulting spot pattern was assigned to each of the three individual 2D-images (Cy2, Cy3, Cy5) of each of the gels in the experiment. For further analysis, uniform spot quantification on all gels and normalization were made according to the volume ratio of corresponding spots detected in the Cy2 image of the pooled internal standard sample using the internal standard module. Student's t-test was performed to assess the statistical significance of differentially expressed proteins. Based on average spot volume ratio, spots whose relative expression is changed at least 2-fold (increase or decrease) between the stagings at 95% confidence level (t-test; p<0.05) were considered to be significant. For subsequent mass spectrometry analysis significant spot coordinates were transferred to Coomassie stained preparative gel for spot picking.

The Hierarchical Clustering and the Principal Component Analysis was performed corresponding to the Delta2D Software protocol: The protein expression data were analyzed by hierarchical clustering to find potential markers which can classify all samples. The hierarchical clustering grouped both, samples (gel images) and expression profiles. Clustering methods grouped expression profiles and gel images by similarity. The cluster hierarchy was shown in a tree display. The cluster composition reflected the structure of the experiment, e.g. replicates and images from the same sample gave similar expression levels and thus ended up in the same cluster. The clustering was performed using all expressed proteins of all 16 samples (quadruple replicates of control, pT1, pT2 and pT3) included in the analysis set.

PCA was performed with the whole set of detected spots of all gel images resulted in a three-dimensional visualization. The information of all samples was linked together by an orthogonal transformation and an overall pattern is presented in form of the principal components.

After identification of proteins by mass spectrometry the significant differentially expressed proteins were analyzed by Ingenuity Pathway Analysis (IPA) to find the shortest pathways between the identified proteins and to constitute molecular partners involved in particular disease. A master global network of all differentially expressed proteins (input objects) was suggested according to published literature-based annotations, and then the further sub networks were built from the master network to focus activated experiments and/or pathways. Major hubs were identified based on the connections and edges with in the network. The heterogeneous set of the identified proteins was also analyzed using gene ontology, which provides information about gene function and cellular localization.

### Preparation of peptide mixtures for MALDI-MS

Protein identification was performed as described earlier [21], [22]. Briefly, proteins were excised from Colloidal Coomassie Brilliant Blue stained 2D gels using a spot cutter (Proteome WorksTM, Biorad, Hercules, CA, USA) with a picker head of 1.5 mm diameter and transferred into 96 well microplates loaded with 100 µl LiChrosolv**^®^** water (Merck KGaA, Darmstadt, Germany) per well. Digestion with trypsin and subsequent spotting of peptide solutions onto the MALDI-targets were performed automatically in the Ettan Spot Handling Workstation (Amersham-Biosciences, Uppsala, Sweden) using a modified standard protocol. Gel pieces were washed twice with 100 µl 50 mM ammoniumbicarbonate/50% (v/v) methanol for 30 min and once with 100 µl 75% (v/v) ACN for 10 min. After drying 10 µl trypsin solution containing 20 ng/µl trypsin (Promega, Madison, WI, USA) in 20 mM ammoniumbicarbonate was added and incubated at 37°C for 120 min. For peptide extraction gel pieces were covered with 60 µl 50% (v/v) ACN/0.1% (w/v) TFA and incubated for 30 min at 37°C. The peptide containing supernatant was transferred into a new microplate and the extraction was repeated with 40 µl of the same solution. The supernatants were dried at 40°C for 220 min completely. Peptides were dissolved in 2.2 µl of 0.5% (w/v) TFA/50% (v/v) ACN and 0.7 µl of this solution were directly spotted on the MALDI-target. Then, 0.4 µl of matrix solution (50% (v/v) ACN/0.5% (w/v) TFA) saturated with α-cyano-4-hydroxycinnamic acid was added and mixed with the sample solution by aspirating the mixture five times. Prior to the measurement in the MALDI-ToF instrument the samples were allowed to dry on the target 10 to 15 min.

### MALDI-MS

The MALDI-MS measurement of spotted peptide solutions was carried out on a 4800 MALDI-ToF/ToF™ Analyzer (Applied Biosystems, Foster City, CA, USA). The spectra were recorded in reflector mode in a mass range from 800 to 4000 Da with a focus mass of 2000 Da. For one main spectrum 25 sub-spectra with 100 shots per sub-spectrum were accumulated using a random search pattern. If the autolytical fragment of trypsin with the mono-isotopic (M+H)+m/z at 2211.104 reached a signal to noise ratio (S/N) of at least 10, an internal calibration was automatically performed using this peak for one-point-calibration. Calibration was performed manually for the less than 1% samples for which the automatic calibration failed. Additionally MALDI-MS/MS analysis was performed for the five strongest peaks of the MS-spectrum after subtraction of peaks corresponding to background or trypsin fragments. For one main spectrum 20 sub-spectra with 125 shots per sub-spectrum were accumulated using a random search pattern. The internal calibration was automatically performed as one-point-calibration if the mono-isotopic arginine (M+H)+m/z at 175.119 or lysine (M+H)+m/z at 147.107 reached a signal to noise ratio (S/N) of at least 5. After calibration a combined database search of MS and MS/MS measurements was performed using the GPS Explorer software (Ver. 3.6, Applied Biosystems, Foster City, CA, USA) with the following settings: (i) MS peak filtering: mass range from 800 to 4000 Da; minimum S/N filter of 10; peak density of 50 peaks per range of 200 Da and maximal 200 peaks per protein spot; mass exclusion list contained background peaks and trypsin fragments with an exclusion tolerance of 50 ppm (ii) MS/MS peak filtering: mass range from 60 Da to a mass that was 20 Da lower than the precursor mass; peak density of 50 peaks per 200 Da and maximal 65 peaks per MS/MS; minimum S/N filter of 10 (iii) database search: precursor tolerance 50 ppm and MS/MS fragment tolerance 0.45 Da. Peak lists were compared with the SwissProt database v56.1 human taxonomy. Peptide mixtures that yielded at least twice a mowse score of 56 for database results were regarded as positive identifications.

### Western blotting

Fifty microgram of protein were loaded per lane and separated by 15% SDS-PAGE followed by transfer onto nitrocellulose membrane. Blocking was carried out in 1×Roti-Block solution (Carl Roth GmbH, Karlsruhe, Germany). Membranes were incubated with rabbit anti-Prohibitin (Abcam plc, Cambridge, UK; 1∶5000), rabbit anti-Peroxiredoxin-3 (self-made, 1∶5000) and rabbit anti-S100-A9 (Abcam plc, Cambridge, UK; 1∶1000) in TBST- 5% BSA solution overnight at 4°C. Excess antibodies were removed by washing with TBST. Incubation with peroxidase-conjugated secondary antibody (anti-rabbit IgG,1∶5000 in 1×Roti-Block) was performed for 1 h at room temperature. After three washes, the reaction was developed by the addition of LumiGLO substrate (Cell Signaling Technology Inc., Danvers, MA, USA). The emitted light was captured on X-ray film (CEA Deutschland, GmbH, Hamburg, Germany). Western blotting for RPLP0 (BIOZOL Diagnostica Vertrieb GmbH, Eching, Germany) was performed to verify equivalent protein loading.

### Immunohistochemistry

Paraffin sections from 22 different samples containing tumor tissue with different gradings (7×G1, 7×G2 and 8×G3) were subjected to immunohistochemistry. The used antibodies were the same as for the Western blotting. The immunohistochemical staining for prohibitin (1∶100) and S100-A9 (1∶100) was performed on a Leica Bond™ system (Leica Mikrosysteme Vertrieb GmbH, Wetzlar, Germany) and for peroxiredoxin-3 (1∶4000) a BenchMark^®^XT (Ventana Medical Systems Inc., Tucson, USA) automated staining system was used. Prior to immunohistochemistry endogenous peroxidase activity was blocked. For prohibitin antigen retrieval was performed by heating (epitope retrieval solution pH 6.0). The primary antibodies were incubated for 16 min (prohibitin and S100-A9) and 32 min (peroxiredoxin-3) respectively. Visualization was performed using the avidin-biotin complex method. Photographs were taken on a Zeiss Axioskop 40 microscope equipped with a Nikon DS-2Mv digital camera.

## Results

### 2D-DIGE analysis and mass spectrometry

We analyzed the proteome of kidney cancer pool specimens from 27 cancer patients (pT1, pT2, pT3) and 9 corresponding control tissues (Table 1) by 2D-DIGE in a pI range of 3.0–10.0 and molecular weight range between 10 kDa and approx. 140 kDa. In this range, 989 spots were clearly detected and analyzed using Delta 2D statistical software tool for differential protein expression. The average abundances of spots were quantified and those with relative changes in abundance greater than two times between at least two tumor stages (up or down) at 95% confidence level (p<0.05) were considered.

**Table 1:** Clinicopathologic characteristics of patient samples.

Patient ID	Gender	Age	pT	pN	M	G
Co1	f	85	2	0	0	2
Co2	m	72	2	0	0	2
Co3	m	80	2	0	0	2
Co4	m	80	2	x	0	2
Co5	m	76	2	0	0	2
Co6	m	90	2	0	0	2
Co7	f	73	2	0	0	2
Co8	m	75	2	x	0	2
Co9	f	69	3a	0	0	2
pT1-1	f	61	1	x	x	1
pT1-2	m	75	1	0	0	2
pT1-3	m	80	1	x	x	1
pT1-4	m	58	1	x	x	2
pT1-5	m	71	1	x	0	2
pT1-6	m	73	1	0	0	2
pT1-7	m	57	1	0	0	1
pT1-8	m	81	1	0	0	2
pT1-9	m	84	1	x	0	1
pT2-1	m	80	2	x	0	2
pT2-2	m	81	2	0	x	3
pT2-3	m	44	2	0	0	2
pT2-4	m	69	2	x	0	2
pT2-5	f	70	2	0	0	2
pT2-6	f	85	2	0	0	2
pT2-7	m	80	2	0	0	2
pT2-8	f	57	2	0	0	1
pT2-9	m	76	2	0	2	1
pT3-1	m	66	3a	1	1	3
pT3-2	f	80	3	1	1	3
pT3-3	m	72	3a	0	0	2
pT3-4	m	50	3b	x	1	3
pT3-5	m	62	3b	0	1	2
pT3-6	m	66	3a	0	0	2
pT3-7	f	77	3a	0	1	2
pT3-8	m	73	3b	2	x	3
pT3-9	f	79	3a	0	0	2

The characteristics of the 9 control samples and 27 kidney cancer included gender, age, TNM status and tumor grading. All samples were obtained from the Department of Urology, University of Greifswald.

219 spots, significantly altered in their abundance among all the samples included in the analysis set, were selected for further identification. Interesting spots were excised from preparative gels for protein identification by tryptic in-gel digestion and MALDI-MS/MS analysis. Following a database search using the acquired MS and MS/MS data 187 different proteins representing 176 spots were identified (Table S1). In several spots were identified the same protein, probably due to post translational modifications. On the other hand, some spots contained more than one protein.

**Figure 1:**
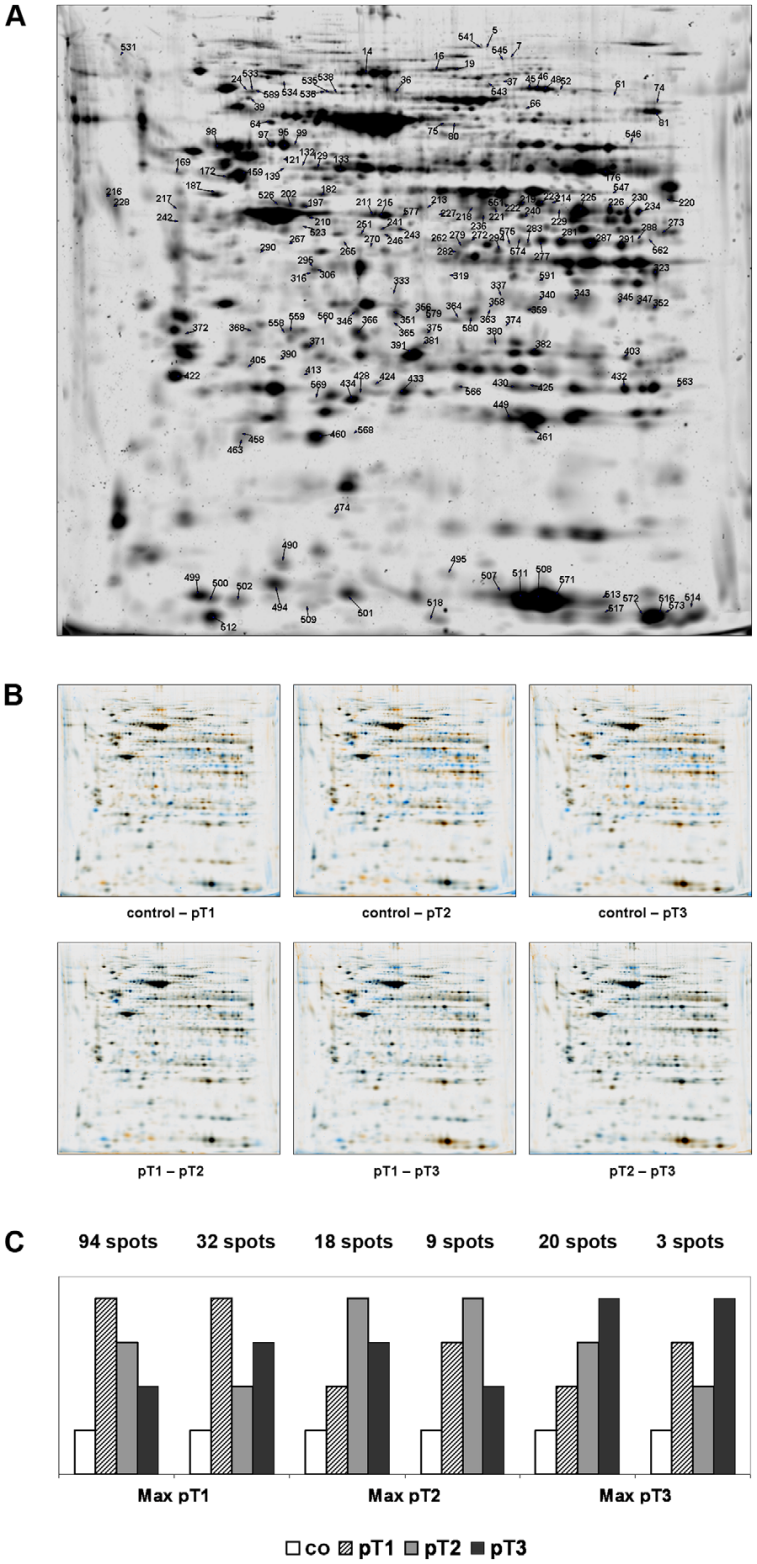
Representative 2-DE proteome map of kidney cancer. **A** 2D proteome map of kidney cancer tissue (fused image), pI range 3–10 NL, 12.5% SDS-PAGE is presented. The identified proteins, which are significantly altered were labeled with numbers. **B** Dual channel image analysis. Differences in spot intensities were analyzed in all possible gel combinations. Each channel represents a fused gel image of all 4 technical replicates. **C** Representation of the possible changes between tumor stages pT1, pT2 and pT3 with different maxima. Above the bars, the number of spots that follow this trend is mentioned.

Exemplarily we issued a proteome map of kidney cancer tissue (fused images, pH 3-10 NL) which was shown in Figure 1A. The numbered spots depict at least 2-fold differentially expressed proteins. The dual channel image analysis showed differences in spot intensities in all possible gel combinations (Figure 1B). Each channel represented a fused gel image of all 4 technical replicates. The differentially expressed spots have been divided into stage related groups like maximum at pT1 (126 spots), maximum at pT2 (27 spots) or maximum at pT3 (23 spots). This principle distribution was shown in Figure 1C illustrating the data from Table S1.

### Classification of Differentially Regulated Proteins

**Figure 2:**
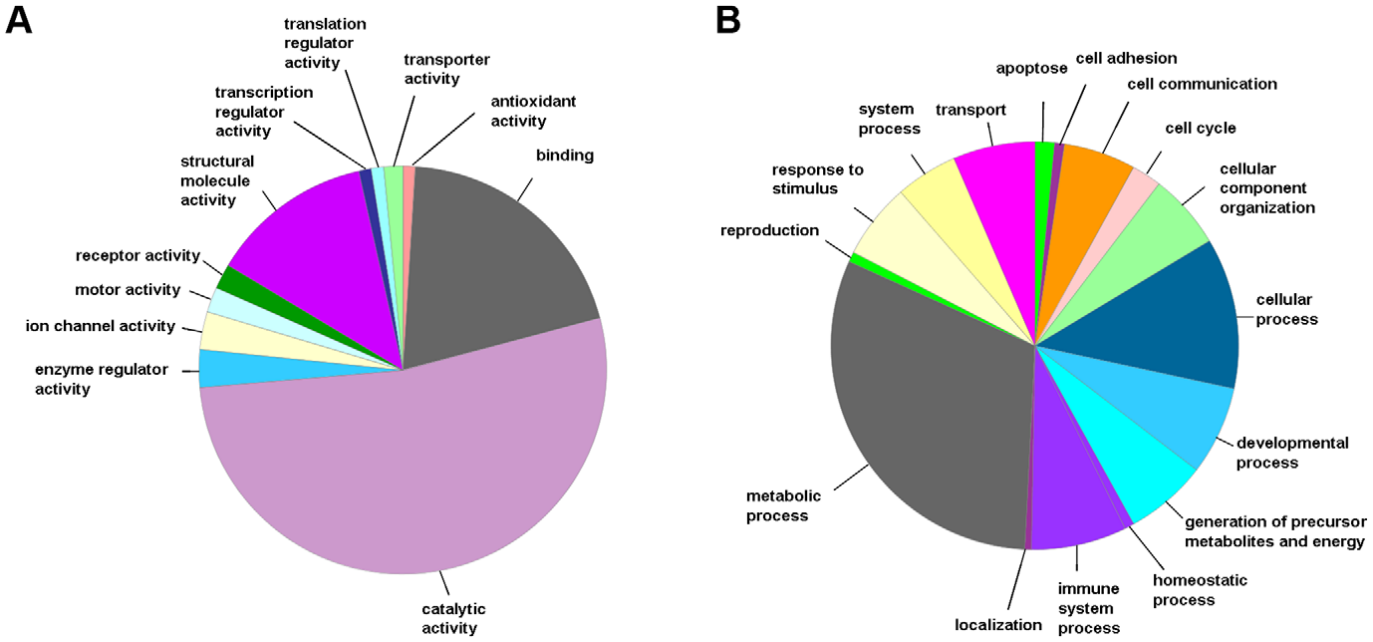
Classification of differentially expressed proteins according to their cellular function. The pie charts display the classification of the subset of differentially expressed proteins listed in Table S1 divided into 11 molecular functional categories (**A**) and into 16 biological processes (**B**). Molecular functions regulated by the differentially expressed proteins classified by Gene Ontology.

In terms of cellular functions and processes, the proteins were distributed into the categories (A) molecular function: ion channel activity (3.3%), transporter activity (1.7%), translation regulator activity (1.1%), transcription regulator activity (1.1%), enzyme regulator activity (3.3%), catalytic activity (58%), motor activity (2.2%), receptor activity (2.2%), antioxidant activity (1.1%), structural molecule activity (14.4%) and binding (22.1%); (B) biological processes: apoptosis (3.3%), transport (13.8%), system process (10.5%), response to stimulus (12.7%), reproduction (1.7%), metabolic process (65.7%), localization (1.1%), immune system process (16%), homeostatic process (1.7%), generation of precursor metabolites and energy (13.8%), developmental process (14.9%), cellular process (25.4%), cellular component organization (12.7%), cell cycle (5%), cell communication (12.2%) and cell adhesion (1.7%) (Figure 2A and 2B).

### Hierarchical clustering and Principal Component Analysis

**Figure 3:**
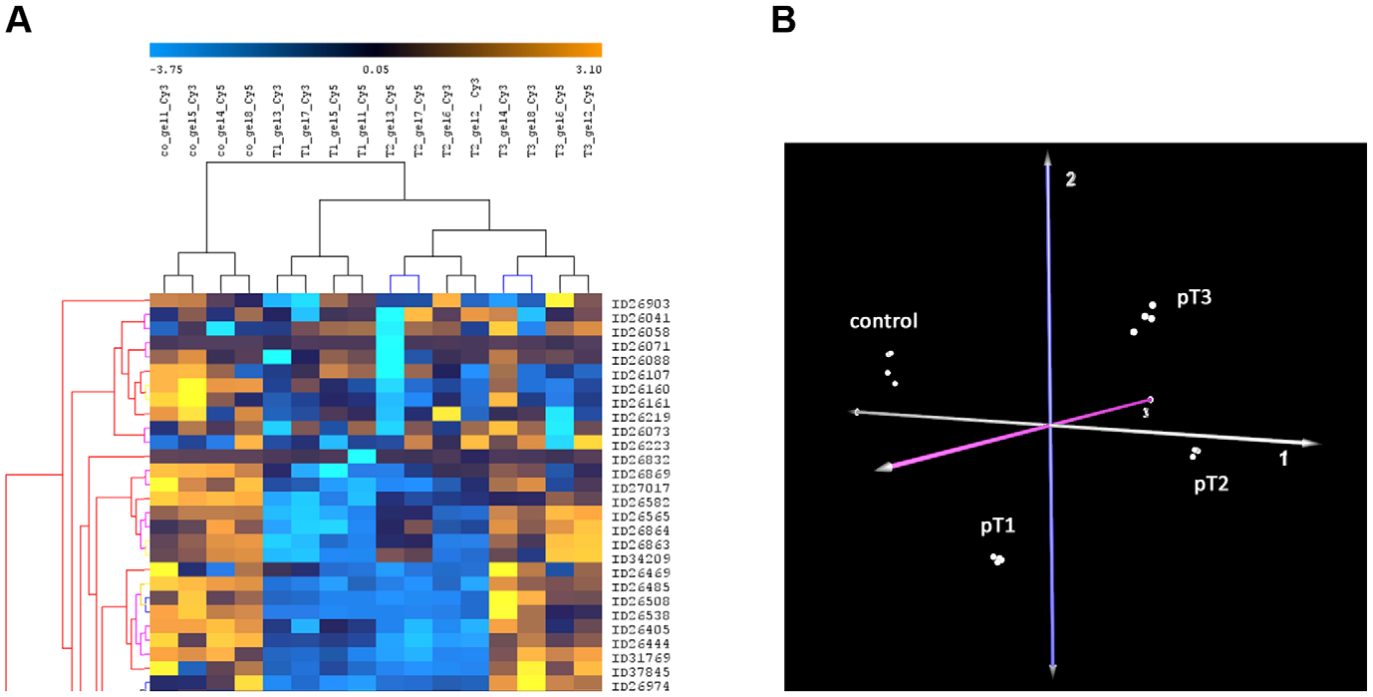
Hierarchical clustering and Principal Component Analysis. Principal component analysis can separate normal and tumor tissue. **A** Partial representation of the unsupervised clustering (Euclidean Distance measure and the ‘average’ linkage) was performed using the log transformed expression protein values of 16 samples (quadruple replicates of control, pT1, pT2 and pT3). The samples are shown vertically, the spots horizontally indicated by ID numbers. The dendrograms represent the distances between the clusters. High expressions are coloured orange, the lower ones in blue. **B** 3D-plot of the first three principal components of PCA from the protein expression data separates all analyzed samples. Each point represents one sample with different replicates and dye swap, distribution of information with respect to differential expression between tumor stages and control tissues. The output of the model indicates the existence of 4 classes differing significantly according to their separability. They fitted to the analyzed sample groups: control, pT1, pT2 and pT3.

The hierarchical clustering showed a comparison of all spot values of all samples against each other and resulted in distribution for all protein spots on first axis and a distribution of samples by similarity on second axis. A section of those heatmap was shown in Figure 3A. The columns represented the different samples (control, pT1, pT2 and pT3 in quadruples) performed in dye swap experiments and the rows indicated analyzed spots (total: 989). The dendrograms represented the distances between the clusters. All sample groups form distinct and separated clusters, respectively. It could be considered that (a) all replicates of the same group were clustering together, (b) the dye swap of the replicates was clustering clearly in between the same group, (c) control samples (co) were clearly separated from tumor samples (pT1, pT2, pT3), and (d) pT1 cluster was separated from pT2/pT3 cluster.

These results had been affirmed also by the Principal Component Analysis. PCA worked by taking spot intensities on every gel image and assembling them into a vector. The gels from different samples would be in separate regions of the resulting diagram. Their coordinates could be analyzed in order to determine which spots were contributing most to the variance, making them candidates for protein identification and biological interpretation. In Figure 3B the first three dimensions of highest variances (principal component 1 = 51.1%, principal component 2 = 15.8%, principal component 3 = 11.9%, summary of the first three components = 78.8%) distinguished all analyzed samples in a very impressive way: again, control samples were separated from all tumor samples as well as a clear separation could be observed in between the tumor stages.

### Networks analysis of identified proteins by Ingenuity Pathway Analysis

Pathways and networks, in that proteins derived from 2D-PAGE using DIGE techniques (differentially expressed in kidney cancer) are involved, were analyzed using Ingenuity Pathway Analysis.

In conclusion of this analysis 187 proteins were classified concerning top biological functions and top networks attached to associated function. Among them 86 proteins related to cancer (p-value 7.79E-14–2.40E-02), 117 to genetic disorder (p-value 1.21E-10–2.40E-02), 79 to neurological disease (p-value 1.21E-10–2.40E-02), 59 to skeletal and muscular disorders (p-value 1.21E-10–2.40E-02) and 41 to gastrointestinal disease (p-value 7.95E-08–2.09E-02).

**Figure 4:**
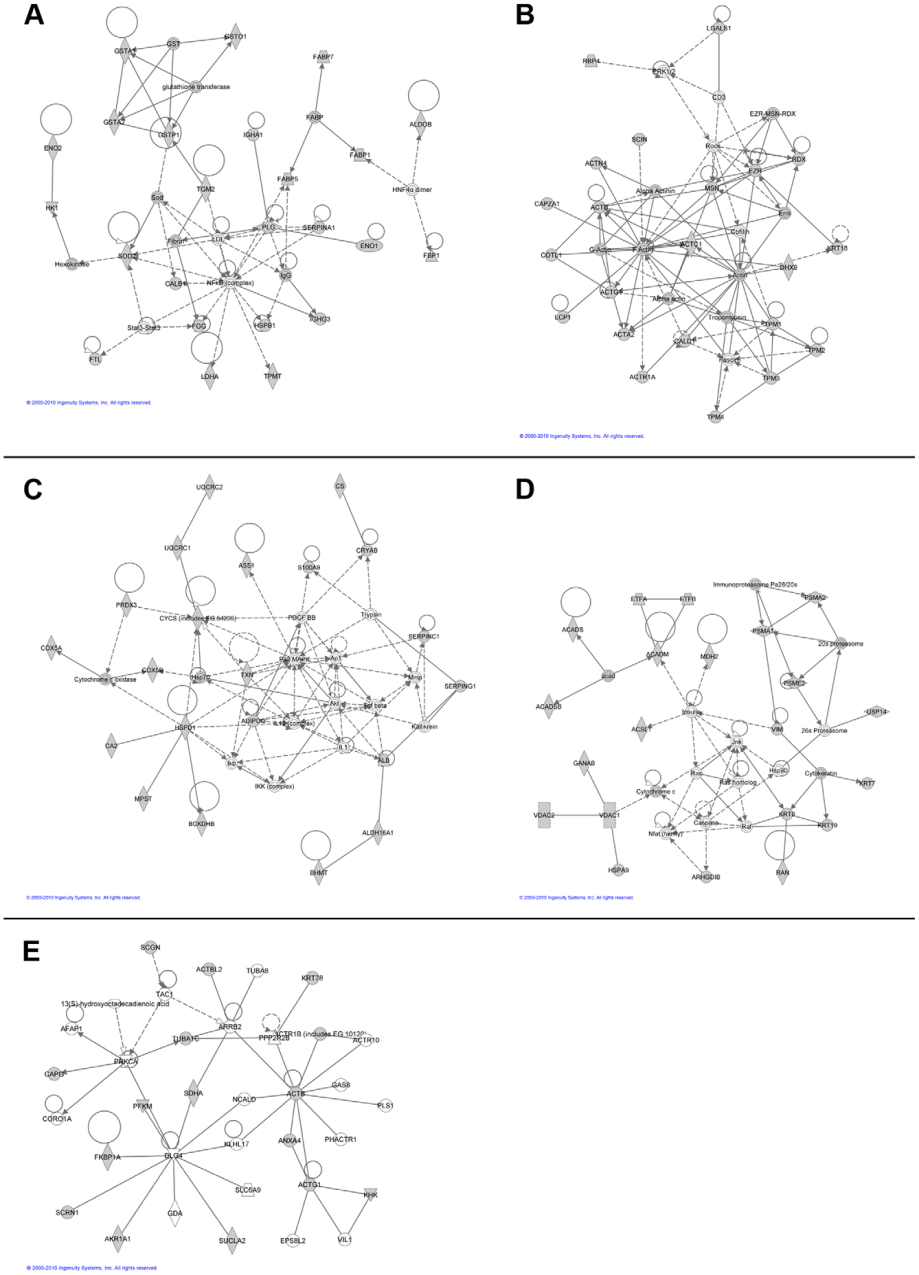
Protein networks of differentially expressed proteins kidney cancer. Ingenuity Pathway Analysis was used to generate a network of direct connections between all identified proteins with altered expression. Grey filled boxes are the differentially expressed proteins. Most significant sub networks were shown in the figure. **A** Lipid Metabolism, Molecular Transport, Small Molecule Biochemistry. 24 proteins out of 36 proteins in the network were confirmed with this analysis **B** Cellular Assembly and Organization, Skeletal and Muscular System Development and Function, Tissue Morphology. 22 proteins out of 33 proteins in the network were confirmed with this analysis. **C** Cellular Movement, Hematopoiesis, Immune Cell Trafficking. 21 proteins out of 35 proteins in the network were confirmed with this analysis. **D** Genetic Disorder, Metabolic Disease, Embryonic Development. 21 proteins out of 34 proteins in the network were confirmed with this analysis. **E** Lipid Metabolism, Small Molecule Biochemistry, Cancer. 16 proteins out of 33 proteins in the network were confirmed with this analysis.

The functional sub-networks were built using IPA from the input proteins (Table 2). The networks would be assigned by a different number of proteins. With identification based on the 2D-DIGE analysis, we found a correlation between the described proteins and our data of around 48%–69%. The most significant sub networks were (A) Lipid Metabolism, Molecular Transport, Small Molecule Biochemistry (24 proteins out of 36 proteins; 69%), (B) Cellular Assembly and Organization, Skeletal and Muscular System, Development and Function, Tissue Morphology (22 proteins out of 33 proteins; 67%), (C) Cellular Movement, Hematopoiesis, Immune Cell Trafficking (21 proteins out of 35 proteins; 60%), (D) Genetic Disorder, Metabolic Disease, Embryonic Development (21 proteins out of 34 proteins; 62%) and (E) Lipid Metabolism, Small Molecule Biochemistry, Cancer (16 proteins out of 33 proteins; 48%) which were represented in Figure 4A–E.

**Table 2:** Ingenuity Pathway Analysis networks of proteomic data and functional protein sub networks altered in kidney cancer.

No^a^	network^b^	described molecules^c^	confirmed with this analysis^d^	%^e^
1	Lipid Metabolism, Molecular Transport, Small Molecule Biochemistry	35	24	69
2	Cellular Assembly and Organization, Skeletal and Muscular System Development and Function, Tissue Morphology	33	22	67
3	Cellular Movement, Hematopoiesis, Immune Cell Trafficking	35	21	60
4	Genetic Disorder, Metabolic Disease, Embryonic Development	34	21	62
5	Lipid Metabolism, Small Molecule Biochemistry, Cancer	33	16	48
6	Post-Translational Modification, Carbohydrate Metabolism, Nucleic Acid Metabolism	34	15	44
7	Lipid Metabolism, Small Molecule Biochemistry, Nucleic Acid Metabolism	31	13	42
8	Cellular Development, Cardiovascular System Development and Function, Cell Morphology	35	13	37
9	Cell Death, Cell Morphology, Connective Tissue Development and Function	34	13	38
10	Lipid Metabolism, Small Molecule Biochemistry, Genetic Disorder	29	12	41
11	Nucleic Acid Metabolism, Small Molecule Biochemistry, DNA Replication, Recombination, and Repair	35	10	29
12	Carbohydrate Metabolism, Cell-To-Cell Signaling and Interaction, Hematological System Development and Function	25	5	20
13	Endocrine System Development and Function, Small Molecule Biochemistry, Lipid Metabolism	2	1	50
14	Cell Cycle, Reproductive System Development and Function, DNA Replication, Recombination, and Repair	3	1	33

**a** network number.

**b**description of the network.

**c**number of described proteins in the network.

**d**number of proteins in the network confirmed by this approach in comparison to the described proteins.

**e**percentage of confirmation.

### Validation of selected candidates by immunohistochemistry and Western blotting

Three candidates were selected for further validation: (a) prohibitin, (b) thioredoxin-dependent peroxide reductase PRDX3 and (c) protein S100-A9.

**Figure 5:**
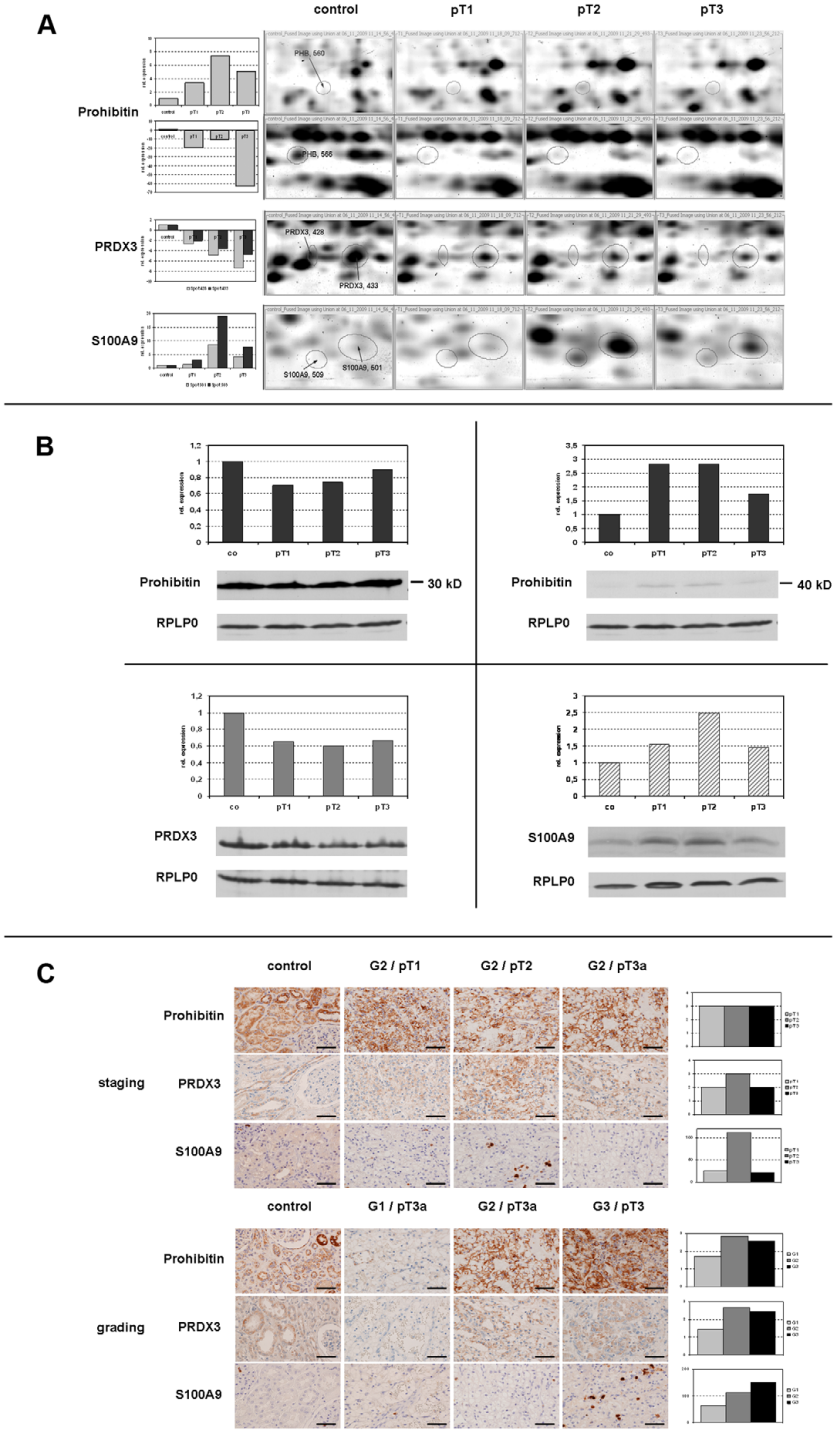
Validation of selected candidates. Prohibitin, thioredoxin-dependent peroxide reductase PRDX3 and protein S100-A9 were analyzed by immunohistochemistry. Western blotting is displayed in comparison to detailed information from the 2D-DIGE. **A** 2D-DIGE snapshots from fused gel images for prohibitin (30 kD and 40 kD), thioredoxin-dependent peroxide reductase PRDX3 and protein S100-A9 with quantification data (bar graph). **B** Protein quantification for prohibitin (30 kD and 40 kD), thioredoxin-dependent peroxide reductase PRDX3 and protein S100-A9 by Western blotting, only representative blots were shown here. RPLP0 was used as loading control. **C** Immunohistochemistry of staging dependencies (pT1, pT2, pT3) of G2 tumors (above). The bars represent semiquantitative quantifications of one representative sample. Immunohistochemistry depending on tumor grade (G1, G2, G3; below). The bars represents semiquantitative quantifications of different samples (7×G1, 7×G2, 8×G3).

In Figure 5A, selected gel regions and the quantification of 2D spots were explicitly presented for the considered candidates. The results from the 2D gel expression patterns were evaluated by using standard Western blot analysis in a one-dimensional gel system. The estimated protein amounts were depicted as columns (Figure 5B). Immunohistochemical assessment with consideration of tumor size (pT1, pT2, pT3) was supplemented by inclusion of tumors belonging to different gradings (7×G1, 7×G2 and 8×G3) (Figure 5C). The intensity of the tumor staining was scored 1 (negative to weak), 2 (intermediate) and 3 (strong). In the case of S100-A9 positive cells were counted in twenty visual fields (Figure 5C, right; Table 3). The evaluation of the control tissue proved to be complicated because of different proportions of glomeruli, tubules and collecting ducts in the selected tissue slices.

**Table 3:** Immunohistochemistry-semiquantitative quantifications of selected candidates depending on tumor grade.

		prohibitin	S100-A9	PRDX3
**G1**		2	57	2
**G1**		2	15	1
**G1**		2	130	2
**G1**		2	38	1
**G1**		1	50	1
**G1**		2	86	1
**G1**		1		2
	mean	**1,71**	**62,67**	**1,43**
**G2**		3	79	2
**G2**		3	60	2
**G2**		3	25	3
**G2**		3	112	3
**G2**		3	22	3
**G2**		2	380	3
**G2**		3		
	mean	**2,86**	**113,00**	**2,67**
**G3**		3	350	3
**G3**		3	280	3
**G3**		3	220	1
**G3**		3	136	3
**G3**		3	154	3
**G3**		3	158	3
**G3**		1	4	1
**G3**		2	103	3
	mean	2,57	150,71	2,43

The intensity of the tumor staining was scored 1 (negative to weak), 2 (intermediate) and 3 (strong). In the case of S100-A9 positive cells were counted in twenty visual fields.

For prohibitin two spots were identified with alternating expression profiles: the spot 560 showed generally a weak expression with a maximum at pT2 whereas spot 566 revealed a decreased expression in all tumor stages in relation to the control. In Western blot analysis the prohibitin antibody detected two bands, whose molecular weight and expression level correlated with the identified 2D spots (Figure 5A, 5B). In immunohistochemistry the total amount of prohibitin seems to be not altered stage-dependently (Figure 5C, upper lane). Additionally we observed in our immunohistochemical studies a considerable grading-dependent increase for total prohibitin.

Also PRDX3 as well as S100-A9 were identified in two spots, each with similar expression profile. In contrast to the strong PRDX3 expression in the control a continuous stage-dependent decrease was observed in tumor samples.

A decrease in relation to the control was also indicated in the Western blot. This diminishment was evident and confirms the 2D data but was not definitely stage-correlated. Considering only the tumor samples, the immunohistochemical estimation of PRDX3 revealed a maximum at pT2 as well as in G2 tumors. Concerning S100-A9 protein the spot pattern displayed a very weak expression in the control accompanied by a maximal expression at pT2, which was also observed in the Western blot and histochemically, whereas the grading dependence was increasing (Figure 5A, B, C).

Immunohistochemical evaluation of the kidney cancer tissue sections exhibited prohibitin as localized in cells of the adjacent renal parenchyma and in glomerular epithelial cells. PRDX3 was detected in the tubulus epithelia with a stronger expression in the distal part of the tubular system. S100-A9 was found in inflammatory cells in blood vessels as well as in the interstitium indicating tumor infiltration (Figure 5C).

## Discussion

In the present study we compared the proteome of non-neoplastic and neoplastic renal tissues. In comparison to our previous studies [7], [8], [13] we now extended the analysis to identify stage-related protein alterations by using 2D-DIGE techniques and network/pathway analysis. Compared to conventional 2DE, DIGE-based proteomics with fluorescence labeling has many advantages such as higher sensitivity, reproducibility with linear dynamic range for better quantification, and less technical variations because of a pooled control as internal standard. Internal controls affirm respectable quality as has been demonstrated impressively by the results of the Principal Component Analysis (Figure 3B).

To the best of our knowledge, we provide a comprehensive map of kidney cancer within stage-related alterations of 187 proteins. Compared to previous literature data [23]–[31], 93 proteins were confirmed (Table S2). Differences between our results and other previously published proteomic analyses could be attributed to technical differences (i.e., the mass spectrometry approaches) and the nature of the material analyzed (*e.g*., cell culture vs. tissue). Other important factors to be considered are tumor heterogeneity and the differences in histological types of the tumors analyzed [27]. Nevertheless we could identify 94 proteins newly described in relation to kidney cancer using the DIGE technique.

We observed in our studies most striking expression differences for proteins involved in remodelling of the cytoskeleton, mitochondrial dysfunctions and changes in lipid metabolism (Table 3, Figure 4). Renal cell cancer mostly arises from the proximal renal tubules. Cells of the proximal tubule rely strongly on mitochondrial function to supply the energy for extensive resorption mediated by Na^+^/K^+^ ATPases in the basolateral membrane. As a typical metabolic pattern these cells produce ATP from fatty acid oxidation and ketone body degradation, but tumorigenesis demands a switch to glycolysis. The fact that tumor cells are characterized by a specific energy metabolism is known for a long time [32]. In the light of the hypothesis of Warburg it was not surprisingly that we found typical enzymes of glycolysis (HK, PFK, TPIS) up-regulated and in contrast enzymes and structural proteins of the respiratory chain (NDUS2, COX5A/B, QCR1/2, CYC, ATPA/B/G) and TCA cycle (CISY, DHSH, IDHP) downregulated (Tabelle S1). These specific metabolic alterations enable the tumor to grow under hypoxic conditions. The benefit for the tumor is the prevention of oxidative stress, because the mitochondrial oxidative phosphorylation is accompanied by the production of reactive oxygen species. Additionally the increasing acidification by high lactate production promotes invasive tumor growth and metastasis [33], [34]. We also observed downregulation of typical β-oxidation enzymes (ECHM, Acyl-CoA-DHG) which confirmed the dramatic metabolic alteration in kidney cancer. The glucose dependency of most tumor cells provides therapeutical approaches like pharmacological inhibition of glycolysis or a carbohydrate reduced/ketogenic diet [35], [36]. On the other hand, antiglycolytic strategies seems to be promising approaches to overcome chemoresistance in cancer cells [37], since it is known that the treatment options in kidney cancer are limited.

An important goal of our study was the comparison of tumor tissues of different stages. We used this experimental strategy to prove whether this procedure is able to identify stage-related, differentially expressed proteins which may be useful as progress markers or are of prognostic value. Considering this and the apparent mitochondrial alterations in kidney cancer we selected for further validation two mitochondrial proteins (prohibitin, PRDX3) which we found as differentially expressed in our 2D-DIGE approach, and the S100-A9 protein. More than 90% of the analyzed samples were classified as G2. Therefore, we additionally tested histopathologically whether the behaviour of our candidates is associated to the grading classification of the tumors as well.

Prohibitin has been functionally linked to diverse processes, such as transcriptional regulation, cell proliferation, development and mitochondrial function [38]–[40].

Prohibitin has been associated with various types of cancer. Initially prohibitin was identified as a tumor suppressor with anti-proliferative potential [41]. The role of prohibitin as tumor-suppressor has still been controversially debated [38], [42], [43]. In breast cancer cells siRNA mediated silencing of prohibitin was found to increase cell proliferation [44]. But the fact that in numerous cancer cells prohibitin has been found to be overexpressed its role as a tumor suppressor remained doubtful [45]. In other studies a prohibitin silencing correlates with reduced cell proliferation and there are indications that prohibitin may function as a cell survival or antiapoptotic factor [46]–[49].

As mentioned in the results we found prohibitin in two different spots by mass spectrometry. Both spots revealed a conversely expression profile. Indeed, the used antibody detected two bands of approximately 30 kDa and 40 kDa, respectively. Most probably the detected band at 40 kDa represents a larger post-translational-modified protein. Many studies have indicated that prohibitin undergoes multiple post-translational modifications, such as phosphorylation, glycosylation, palmitoylation, ubiquitination and others [50]–[52]. Such modifications could explain the shift in molecular weight of the two spots and admits to speculate about the consequences.

Work is in progress to identify the nature of this modification and its stage-correlated physiological role. Considering only the tumor staging the total amount of prohibitin did not seem to be changed immunohistochemically. The fact that the prohibitin antibody detects both forms (30 and 40 kDa) explains these findings. Since we observed a grading-dependent increase for total prohibitin, it could gain importance as a progression marker.

Alterations represented in network 3 (Figure 4C) including PRDX3 and S100-A9 which were also selected for further validation. PRDX3 is involved in redox regulation of the cell and protects cells against oxidative stress-associated apoptosis, as has been shown previously [53], [54]. PRDX3 belongs to a protein family of thiol-specific antioxidative enzymes whose members are involved in the enzymatic degradation of hydrogen peroxide, organic hydroperoxides, and peroxynitrite [53]. Oxidative stress caused by reactive oxygen species is considered to have tumorigenic potential. Aberrant expression of peroxiredoxins was found in a variety of cancer entities [55]. Especially for PRDX3 increased expression was found in human breast cancer, lung cancer, mesothelioma and hepatocellular carcinomas [56]–[59]. In the present study we could demonstrate a significant stage-related downregulation of PRDX3 in tumor cells compared to non-neoplastic tissue (Figure 5A). But there was no association with tumor size in the Western blot (Figure 5B). Immunohistochemical studies however weakly suggested a correlation to size and grading (Figure 5C). Considering this PRDX3 showed only less potential to become a progress marker. This is in agreement with an earlier study where the authors found that only 23% of kidney cancer revealed positive immunoreactivity for PRDX3 [60].

The S100-A9 protein is a member of the S100 protein family, a group of multi-genic, calcium-binding proteins that are differentially expressed in a wide variety of cell types [61]. S100-A9 is mainly expressed by macrophages in acutely inflamed tissues and in chronic inflammation, detected in peripheral blood leukocytes, in neutrophils and granulocytes infiltrating the tumor tissue. Increased S100-A9 levels were detected in various human cancers, presenting abundant expression in neoplastic tumor cells as well as infiltrating immune cells [62]–[65]. In addition to other proposed functions, S100-A9 promotes NF-κB activation and has been associated with tumor development, cancer invasion or metastasis [61]. Mice lacking S100-A9 showed no obvious phenotype, they are viable und fertile [66], [67], but in a mouse model of colon cancer they showed significantly reduced tumor incidence, growth and metastasis [68]. If this could be confirmed in a renal cancer model, S100-A9 would be a promising target to study tumor progression. We observed a stage- and clearly grading-associated increase of S100-A9. Nevertheless, further studies need to be conducted to validate S100-A9 as a marker for tumor infiltration or as a possible candidate with prognostic predictions concerning tumor development.

In conclusion, the here established, comprehensive kidney cancer map depicting stage-related alterations extends the list of disease-associated proteins. Some of these candidates might be the starting point for further research to understand tumor development and to develop diagnostic or prognostic markers. For that purpose a complex proteome approach using 2D-DIGE combined with an extensive statistical data analysis and subsequent principal component analysis has been shown to be appropriate.

## Supporting Information

Table S1 **Differentially expressed proteins identified in kidney cancer sample.** Stage-related alterations in kidney cancer-Identification of protein targets with 2D-DIGE coupled mass spectrometry. No. at Figure 1, spot ID on consensus master 2D gel; DB accession*, official SwissProt accession number; Protein name; Protein Score; Ratio, ratio pT1 /control, ratio pT2 /control, ratio pT3 /control; Trends, max pT1 with 2 subgroups, max pT2 with 2 subgroups, max pT3 with 2 subgroups, x = mapping corresponds to the groups; Protein MW, theoretical mass; Protein PI, theoretical isoelectric point; Peptide Count, total number of peptides for identification using mass spectrometry; Sequence coverage, sequence covarage in percent; Sequence coverage ms ms, sequence coverage in percent defined by MS/MS data; Sequence coverage ms ms sig, sequence coverage in percent defined by significant MS/MS data; Ions score; Peptides with ions score, number of sequenced peptides with an ion score; Peptides above identity threshold, number of peptides above identity threshold; Peptides above homology threshold, number of peptides above homology threshold; Rms error, peptide mass error tolerance. SwissProt database v56.1 human taxonomy; cut off score>56 with p-value<0.05, search parameters: MS/MS ion search, enzyme: trypsin, variable modifications: carbamidomethyl (C), oxidation (M), peptide mass tolerance: ±50 ppm, fragment mass tolerance: ±0.45 Da, max missed cleavages: 1. *all entries contain _HUMAN extension.
(XLS)

Table S2 **Comparison of the identified proteins from the 2D-DIGE experiment with published data.** All proteins were listed by name. Different references were given, marked by reference number. The last rows include a summary of earlier described proteins and separate them from newly detected and identified proteins by this approach. *DB accession: according to SwissProt (all entries contain _HUMAN extension).
(DOC)
