# Supplementary material for: Stage-Related Alterations in Renal Cell Carcinoma – Comprehensive Quantitative Analysis by 2D-DIGE and Protein Network Analysis
Source: PLoS One. 2011 Jul 7;6(7):e21867. doi: 10.1371/journal.pone.0021867 (PMC3131398; doi:10.1371/journal.pone.0021867)
Supplement: Table S2 — Comparison of the identified proteins from the 2D-DIGE experiment with published data. All proteins were listed by name. Different references were given, marked by reference number. The last rows include a summary of earlier described proteins and separate them from newly detected and identified proteins by this approach. *DB accession: according to SwissProt (all entries contain _HUMAN extension). (DOC) [file pone.0021867.s002.doc]

**Table S2: Comparison of the identified proteins from the 2D-DIGE experiment with published data. All proteins were listed by name. Different references were given, marked by reference number. The last rows include a summary of earlier described proteins and separate them from newly detected and identified proteins by this approach. *DB accession: according to SwissProt (all entries contain _HUMAN extension)**

| **DB accession*** | **Protein Name** | **Kim *et al. [23]*** | **Johann *et al. [24]*** | **Lichtenfels *et al. [25]*** | **Seliger *et al. [26]*** | **Siu *et al. [27]*** | **Okamura *et al. [28]*** | **Seliger review *et al. [29]*** | **Perroud *et al. [30]*** | **Craven *et al. [31]*** | **no of previously described** |
| --- | --- | --- | --- | --- | --- | --- | --- | --- | --- | --- | --- |
| **3HIDH** | 3-hydroxyisobutyrate dehydrogenase, mitochondrial |  |  | x |  |  |  |  |  |  | 1 |
| **A16A1** | Aldehyde dehydrogenase family 16 member A1 |  |  |  |  |  |  |  |  |  | new |
| **A1AG1** | Alpha-1-acid glycoprotein 1 |  | x |  |  |  |  |  |  |  | 1 |
| **A1AT** | Alpha-1-antitrypsin |  | x |  |  |  |  |  |  |  | 1 |
| **A1BG** | Alpha-1B-glycoprotein |  |  |  |  |  |  |  |  |  | new |
| **A26CA** | ANKRD26-like family C member 1A |  |  |  |  |  |  |  |  |  | new |
| **A26CB** | ANKRD26-like family C member 1B |  |  |  |  |  |  |  |  |  | new |
| **AATM** | Aspartate aminotransferase, mitochondrial |  |  |  |  |  |  |  |  |  | new |
| **ACADM** | Medium-chain specific acyl-CoA dehydrogenase, mitochondrial |  |  | x |  | x |  |  |  |  | 2 |
| **ACADS** | Short-chain specific acyl-CoA dehydrogenase, mitochondrial |  |  | x |  |  |  |  |  |  | 1 |
| **ACDSB** | Short/branched chain specific acyl-CoA dehydrogenase, mitochondrial |  |  |  |  |  |  |  |  |  | new |
| **ACON** | Aconitate hydratase, mitochondrial |  |  | x |  |  | x |  |  |  | 2 |
| **ACOT1** | Acyl-coenzyme A thioesterase 1 |  |  |  |  |  |  |  |  |  | new |
| **ACOT2** | Acyl-coenzyme A thioesterase 2, mitochondrial |  |  |  |  |  | x |  |  |  | 1 |
| **ACSL1** | Long-chain-fatty-acid--CoA ligase 1 |  |  |  |  |  |  |  |  |  | new |
| **ACTA** | Actin, aortic smooth muscle |  |  |  |  |  |  |  |  |  | new |
| **ACTB** | Actin, cytoplasmic 1 |  |  |  |  |  |  |  |  |  | new |
| **ACTBL** | Beta-actin-like protein 2 |  |  |  |  |  |  |  |  |  | new |
| **ACTC** | Actin, alpha cardiac muscle 1 |  |  |  |  |  |  |  |  |  | new |
| **ACTG** | Actin, cytoplasmic 2 |  |  | x | x |  |  |  |  | x | 3 |
| **ACTK** | Kappa-actin |  |  |  |  |  |  |  |  |  | new |
| **ACTY** | Beta-centractin |  |  |  |  |  |  |  |  |  | new |
| **ACTZ** | Alpha-centractin |  |  |  |  |  |  |  |  |  | new |
| **ACY1** | Aminoacylase-1 |  |  |  |  |  | x |  | x |  | 2 |
| **ADIPO** | Adiponectin |  |  |  |  |  |  |  |  |  | new |
| **ADSV** | Adseverin |  |  |  |  |  |  |  |  |  | new |
| **AK1A1** | Alcohol dehydrogenase [NADP+] |  |  | x |  |  |  |  |  | x | 2 |
| **ALBU** | Serum albumin |  |  |  |  |  |  |  |  |  | new |
| **ALDOB** | Fructose-bisphosphate aldolase B |  |  | x | x |  | x | x | x |  | 5 |
| **ANT3** | Antithrombin-III |  |  |  |  |  |  |  |  |  | new |
| **ANXA4** | Annexin A4 |  |  | x | x | x | x | x | x |  | 6 |
| **ARPC5** | Actin-related protein 2/3 complex subunit 5 |  |  |  |  |  |  |  |  |  | new |
| **ASSY** | Argininosuccinate synthase |  |  |  |  |  |  |  | x |  | 1 |
| **ATPA** | ATP synthase subunit alpha, mitochondrial |  |  | x |  |  |  |  | x |  | 2 |
| **ATPB** | ATP synthase subunit beta, mitochondrial |  |  |  |  | x |  |  |  |  | 1 |
| **ATPG** | ATP synthase subunit gamma, mitochondrial |  |  |  |  |  |  |  |  |  | new |
| **BHMT1** | Betaine--homocysteine S-methyltransferase 1 |  |  |  |  |  | x |  | x |  | 2 |
| **BHMT2** | Betaine--homocysteine S-methyltransferase 2 |  |  |  |  |  |  |  |  |  | new |
| **BPHL** | Valacyclovir hydrolase |  |  |  |  |  |  |  |  |  | new |
| **CAH1** | Carbonic anhydrase 1 |  |  |  |  |  |  |  |  |  | new |
| **CAH2** | Carbonic anhydrase 2 |  |  | x |  |  |  |  |  |  | 1 |
| **CALB1** | Calbindin |  |  | x |  |  |  |  | x |  | 2 |
| **CALB2** | Calretinin |  |  |  |  | x |  |  |  |  | 1 |
| **CALD1** | Caldesmon |  |  |  |  |  |  |  |  |  | new |
| **CAPG** | Macrophage-capping protein | x |  |  |  |  |  |  |  |  | 1 |
| **CATD** | Cathepsin D |  |  |  |  |  |  |  |  | x | 1 |
| **CAZA1** | F-actin-capping protein subunit alpha-1 |  |  |  |  |  |  |  |  |  | new |
| **CH60** | 60 kDa heat shock protein, mitochondrial |  |  | x | x | x |  | x | x | x | 6 |
| **CISY** | Citrate synthase, mitochondrial |  |  |  |  |  |  |  |  |  | new |
| **COTL1** | Coactosin-like protein |  |  |  |  |  |  |  |  |  | new |
| **COX5A** | Cytochrome c oxidase subunit 5A, mitochondrial |  |  | x |  |  |  |  |  |  | 1 |
| **COX5B** | Cytochrome c oxidase subunit 5B, mitochondrial |  |  |  |  |  |  |  |  |  | new |
| **CRYAB** | Alpha-crystallin B chain |  |  | x |  | x |  |  |  |  | 2 |
| **CYC** | Cytochrome c |  |  |  |  | x |  |  |  |  | 1 |
| **D3D2** | 3,2-trans-enoyl-CoA isomerase, mitochondrial |  |  |  |  |  |  |  |  |  | new |
| **DECR** | 2,4-dienoyl-CoA reductase, mitochondrial |  |  |  |  | x |  |  |  |  | 1 |
| **DHSA** | Succinate dehydrogenase [ubiquinone] flavoprotein subunit, mitochondrial |  |  | x |  |  | x |  |  |  | 2 |
| **DHSO** | Sorbitol dehydrogenase |  |  |  |  |  |  |  |  |  | new |
| **DHX9** | ATP-dependent RNA helicase A |  |  |  |  |  |  |  |  |  | new |
| **ECH1** | Delta(3,5)-Delta(2,4)-dienoyl-CoA isomerase, mitochondrial |  |  |  |  |  |  |  |  |  | new |
| **ECHA** | Trifunctional enzyme subunit alpha, mitochondrial |  |  |  |  |  |  |  |  |  | new |
| **ECHB** | Trifunctional enzyme subunit beta, mitochondrial |  |  |  |  |  | x |  |  |  | 1 |
| **ECHM** | Enoyl-CoA hydratase, mitochondrial |  |  | x | x |  | x | x |  |  | 4 |
| **ECHP** | Peroxisomal bifunctional enzyme |  |  |  |  |  |  |  |  |  | new |
| **EFTU** | Elongation factor Tu, mitochondrial |  |  |  | x | x |  |  |  |  | 2 |
| **ENOA** | Alpha-enolase |  |  | x | x |  | x |  |  | x | 4 |
| **ENOG** | Gamma-enolase | x |  | x | x |  |  |  | x |  | 4 |
| **ES1** | ES1 protein homolog, mitochondrial |  |  |  |  |  |  |  |  |  | new |
| **ESTD** | S-formylglutathione hydrolase |  |  |  |  |  |  |  |  |  | new |
| **ETFA** | Electron transfer flavoprotein subunit alpha, mitochondrial |  |  |  |  |  |  |  |  |  | new |
| **ETFB** | Electron transfer flavoprotein subunit beta |  |  |  |  |  |  |  |  |  | new |
| **EZRI** | Ezrin |  |  |  | x |  | x |  |  |  | 2 |
| **F16P1** | Fructose-1,6-bisphosphatase 1 |  |  | x |  |  |  |  |  |  | 1 |
| **FABP5** | Fatty acid-binding protein, epidermal |  |  |  |  |  |  |  |  |  | new |
| **FABP7** | Fatty acid-binding protein, brain |  |  | x | x | x |  | x | x |  | 5 |
| **FABPL** | Fatty acid-binding protein, liver |  |  | x | x |  |  | x |  |  | 3 |
| **FIBG** | Fibrinogen gamma chain |  | x | x |  |  | x |  |  |  | 2 |
| **FKB1A** | Peptidyl-prolyl cis-trans isomerase FKBP1A |  |  |  |  |  |  |  |  | x | 1 |
| **FRIL** | Ferritin light chain | x |  |  |  |  |  |  |  |  | 1 |
| **GALM** | Aldose 1-epimerase |  |  |  |  |  | x |  |  |  | 1 |
| **GANAB** | Neutral alpha-glucosidase AB |  |  |  |  |  |  |  |  |  | new |
| **GATM** | Glycine amidinotransferase, mitochondrial |  |  | x |  |  | x |  | x |  | 3 |
| **GBB1** | Guanine nucleotide-binding protein G(I)/G(S)/G(T) subunit beta-1 |  |  |  |  |  |  |  |  |  | new |
| **GDIR2** | Rho GDP-dissociation inhibitor 2 |  |  | x |  |  |  |  |  |  | 1 |
| **GPX3** | Glutathione peroxidase 3 |  | x | x |  |  |  |  |  |  | 2 |
| **GRL1A** | Protein GRINL1A |  |  |  |  |  |  |  |  |  | new |
| **GRP75** | Stress-70 protein, mitochondrial |  |  | x | x |  |  |  |  |  | 2 |
| **GSTA1** | Glutathione S-transferase A1 |  |  |  | x |  |  |  |  |  | 1 |
| **GSTA2** | Glutathione S-transferase A2 |  |  |  |  | x |  |  |  |  | 1 |
| **GSTO1** | Glutathione transferase omega-1 |  |  |  |  | x |  |  | x |  | 2 |
| **GSTP1** | Glutathione S-transferase P |  |  |  | x |  |  |  |  |  | 1 |
| **HBA** | Hemoglobin subunit alpha |  |  |  |  |  |  |  |  |  | new |
| **HBB** | Hemoglobin subunit beta |  |  |  |  | x |  |  |  |  | 1 |
| **HBD** | Hemoglobin subunit delta |  |  |  |  |  |  |  |  |  | new |
| **HCDH** | Hydroxyacyl-coenzyme A dehydrogenase, mitochondrial |  |  | x |  |  |  |  |  |  | 1 |
| **HDHD2** | Haloacid dehalogenase-like hydrolase domain-containing protein 2 |  |  |  |  |  |  |  |  |  | new |
| **HEBP1** | Heme-binding protein 1 |  |  |  |  |  |  |  |  |  | new |
| **HIBCH** | 3-hydroxyisobutyryl-CoA hydrolase, mitochondrial |  |  |  |  |  |  |  |  |  | new |
| **HMGB2** | High mobility group protein B2 |  |  |  |  |  |  |  |  |  | new |
| **HRG** | Histidine-rich glycoprotein |  | x |  |  |  |  |  |  |  | 1 |
| **HSPB1** | Heat shock protein beta-1 | x |  | x | x |  | x |  | x | x | 6 |
| **HXK1** | Hexokinase-1 |  |  |  |  |  |  |  |  |  | new |
| **IC1** | Plasma protease C1 inhibitor |  |  |  |  |  |  |  |  |  | new |
| **IDH3A** | Isocitrate dehydrogenase [NAD] subunit alpha, mitochondrial |  |  |  |  |  | x |  |  |  | 1 |
| **IDHP** | Isocitrate dehydrogenase [NADP], mitochondrial |  |  |  |  |  |  |  |  |  | new |
| **IGHA1** | Ig alpha-1 chain C region |  |  |  |  |  |  |  |  |  | new |
| **IGHA2** | Ig alpha-2 chain C region |  |  |  |  |  |  |  |  |  | new |
| **IGHG3** | Ig gamma-3 chain C region |  |  |  |  |  |  |  |  |  | new |
| **IMMT** | Mitochondrial inner membrane protein |  |  | x |  |  |  |  |  |  | 1 |
| **K1C18** | Keratin, type I cytoskeletal 18 |  |  |  |  |  |  |  |  |  | new |
| **K1C19** | Keratin, type I cytoskeletal 19 |  |  |  |  |  |  |  |  |  | new |
| **K2C7** | Keratin, type II cytoskeletal 7 |  |  |  |  |  |  |  |  |  | new |
| **K2C78** | Keratin, type II cytoskeletal 78 |  |  |  |  |  |  |  |  |  | new |
| **K2C8** | Keratin, type II cytoskeletal 8 |  |  | x | x |  |  | x |  |  | 3 |
| **K6PF** | 6-phosphofructokinase, muscle type |  |  |  |  |  |  |  |  |  | new |
| **K6PP** | 6-phosphofructokinase type C |  |  |  |  |  |  |  |  |  | new |
| **KCD12** | BTB/POZ domain-containing protein KCTD12 |  |  |  |  |  |  |  |  |  | new |
| **KCRB** | Creatine kinase B-type |  |  |  |  |  | x |  |  |  | 1 |
| **KHK** | Ketohexokinase |  |  | x |  |  |  |  |  |  | 1 |
| **KU86** | ATP-dependent DNA helicase 2 subunit 2 |  |  |  |  |  |  |  |  | x | 1 |
| **LACB2** | Beta-lactamase-like protein 2 |  |  |  |  |  |  |  |  |  | new |
| **LDHA** | L-lactate dehydrogenase A chain |  |  |  | x | x | x |  | x | x | 5 |
| **LEG1** | Galectin-1 |  |  |  |  |  | x |  |  |  | 1 |
| **MDHM** | Malate dehydrogenase, mitochondrial |  |  |  |  |  |  |  |  |  | new |
| **MOES** | Moesin |  |  | x | x |  | x |  |  | x | 4 |
| **NDUS2** | NADH dehydrogenase [ubiquinone] iron-sulfur protein 2, mitochondrial |  |  | x |  |  |  |  |  |  | 1 |
| **NHERF** | Ezrin-radixin-moesin-binding phosphoprotein 50 |  |  |  |  |  |  |  |  |  | new |
| **NIT2** | Nitrilase homolog 2 |  |  |  |  |  |  |  |  |  | new |
| **NNMT** | Nicotinamide N-methyltransferase | x |  |  | x | x |  |  |  |  | 3 |
| **NQO2** | Ribosyldihydronicotinamide dehydrogenase [quinone] |  |  | x |  |  |  |  |  |  | 1 |
| **OAT** | Ornithine aminotransferase, mitochondrial |  |  |  |  |  |  |  |  |  | new |
| **ODBB** | 2-oxoisovalerate dehydrogenase subunit beta, mitochondrial |  |  |  |  |  |  |  |  |  | new |
| **ODO1** | 2-oxoglutarate dehydrogenase E1 component, mitochondrial |  |  |  |  |  |  |  |  |  | new |
| **ODPB** | Pyruvate dehydrogenase E1 component subunit beta, mitochondrial |  |  |  | x | x |  |  |  |  | 2 |
| **OGDHL** | 2-oxoglutarate dehydrogenase E1 component-like, mitochondrial |  |  |  |  |  |  |  |  |  | new |
| **P5CS** | Delta-1-pyrroline-5-carboxylate synthetase |  |  |  |  |  |  |  |  |  | new |
| **PBLD** | Phenazine biosynthesis-like domain-containing protein |  |  |  |  |  |  |  |  |  | new |
| **PECI** | Peroxisomal 3,2-trans-enoyl-CoA isomerase |  |  |  |  |  |  |  |  |  | new |
| **PHB** | Prohibitin |  |  |  |  |  |  |  |  |  | new |
| **PLMN** | Plasminogen |  | x |  |  |  |  |  |  |  | 1 |
| **PLSL** | Plastin-2 |  | x |  |  |  |  |  |  |  | 1 |
| **PNCB** | Nicotinate phosphoribosyltransferase |  |  |  |  |  |  |  |  | x | 1 |
| **PNPH** | Purine nucleoside phosphorylase |  |  |  |  |  |  |  |  |  | new |
| **PRDX3** | Thioredoxin-dependent peroxide reductase, mitochondrial |  |  |  |  |  |  |  |  |  | new |
| **PSA1** | Proteasome subunit alpha type-1 |  |  |  |  |  |  |  |  |  | new |
| **PSA2** | Proteasome subunit alpha type-2 |  |  |  |  |  |  |  |  |  | new |
| **PSME2** | Proteasome activator complex subunit 2 |  |  |  |  |  |  |  | x |  | 1 |
| **PYGB** | Glycogen phosphorylase, brain form |  |  |  |  |  |  |  |  | x | 1 |
| **PYGL** | Glycogen phosphorylase, liver form |  |  |  |  |  |  |  |  |  | new |
| **QCR1** | Cytochrome b-c1 complex subunit 1, mitochondrial |  |  |  | x | x | x |  |  |  | 3 |
| **QCR2** | Cytochrome b-c1 complex subunit 2, mitochondrial |  |  |  | x |  |  | x |  |  | 2 |
| **RADI** | Radixin |  |  |  | x |  |  |  |  | x | 2 |
| **RAN** | GTP-binding nuclear protein Ran |  |  |  |  |  |  |  |  |  | new |
| **RCN1** | Reticulocalbin-1 |  |  |  |  | x | x |  |  |  | 2 |
| **RET4** | Retinol-binding protein 4 |  |  | x |  |  | x |  |  |  | 2 |
| **S10A9** | Protein S100-A9 |  |  |  |  |  |  |  |  |  | new |
| **SAHH** | Adenosylhomocysteinase |  |  | x |  |  |  |  |  |  | 1 |
| **SARDH** | Sarcosine dehydrogenase, mitochondrial |  |  |  |  |  |  |  |  |  | new |
| **SCRN1** | Secernin-1 |  |  |  |  |  |  |  |  |  | new |
| **SEC13** | Protein SEC13 homolog |  |  |  |  |  |  |  |  |  | new |
| **SEGN** | Secretagogin | x |  |  |  |  |  |  |  |  | 1 |
| **SERPH** | Serpin H1 |  |  |  |  |  |  |  |  |  | new |
| **SND1** | Staphylococcal nuclease domain-containing protein 1 |  |  |  |  |  |  |  |  |  | new |
| **SODM** | Superoxide dismutase [Mn], mitochondrial |  |  |  | x |  |  | x |  |  | 2 |
| **SORCN** | Sorcin |  |  |  |  |  |  |  |  |  | new |
| **SPEB** | Agmatinase, mitochondrial |  |  | x | x |  |  | x | x |  | 4 |
| **STML2** | Stomatin-like protein 2 |  |  |  |  |  |  |  |  |  | new |
| **SUCB1** | Succinyl-CoA ligase [ADP-forming] subunit beta, mitochondrial |  |  |  |  |  |  |  |  | x | 1 |
| **TBA1C** | Tubulin alpha-1C chain | x |  |  | x |  |  | x |  |  | 3 |
| **TGM2** | Protein-glutamine gamma-glutamyltransferase 2 |  |  | x |  |  |  |  |  |  | 1 |
| **THIM** | 3-ketoacyl-CoA thiolase, mitochondrial |  |  |  |  |  |  |  |  |  | new |
| **THIO** | Thioredoxin |  |  | x | x |  |  | x |  |  | 3 |
| **THNS1** | Threonine synthase-like 1 |  |  |  |  |  |  |  |  |  | new |
| **THTM** | 3-mercaptopyruvate sulfurtransferase |  |  |  |  |  |  |  |  |  | new |
| **THTR** | Thiosulfate sulfurtransferase |  |  |  |  |  | x |  |  |  | 1 |
| **TPIS** | Triosephosphate isomerase |  |  |  | x |  | x | x |  | x | 4 |
| **TPM1** | Tropomyosin alpha-1 chain |  |  |  |  | x |  |  |  |  | 1 |
| **TPM2** | Tropomyosin beta chain |  |  | x |  |  |  |  |  |  | 1 |
| **TPM3** | Tropomyosin alpha-3 chain |  |  |  | x |  |  | x |  |  | 2 |
| **TPM4** | Tropomyosin alpha-4 chain |  | x | x | x |  |  |  |  |  | 3 |
| **TPMT** | Thiopurine S-methyltransferase |  |  |  |  |  |  |  |  |  | new |
| **TYPH** | Thymidine phosphorylase |  |  | x | x |  |  | x |  |  | 3 |
| **UBP14** | Ubiquitin carboxyl-terminal hydrolase 14 |  |  |  |  |  |  |  |  |  | new |
| **UCHL1** | Ubiquitin carboxyl-terminal hydrolase isozyme L1 |  |  |  | x |  |  | x |  | x | 3 |
| **VDAC1** | Voltage-dependent anion-selective channel protein 1 |  |  |  |  |  | x |  |  |  | 1 |
| **VDAC2** | Voltage-dependent anion-selective channel protein 2 |  |  |  |  |  |  |  |  |  | new |
| **VIME** | Vimentin |  |  | x | x | x | x | x |  | x | 6 |
|  |  |  |  |  |  |  |  |  |  |  | 94 new |
